# Survival and Risk Comparison of *Campylobacter jejuni* on Various Processed Meat Products

**DOI:** 10.3390/ijerph13060580

**Published:** 2016-06-09

**Authors:** Soo Hyeon Hong, Han Sol Kim, Ki Sun Yoon

**Affiliations:** Department of Food and Nutrition, College of Human Ecology, Kyung Hee University, 26 Kyunghee-daero, Dongdaemun-gu, Seoul 130-701, Korea; witty1003@gmail.com (S.H.H.); iaol10042@naver.com (H.S.K.)

**Keywords:** *Campylobacter jejuni*, processed meat products, risk assessment

## Abstract

The objective of this study was to investigate survival kinetics of *Campylobacter jejuni* on various processed meat products (dry-cured ham, round ham with/without sodium nitrite, garlic seasoned ham with/without sodium nitrite, and sausage without sodium nitrite). Additionally, a semi-quantitative risk assessment of *C. jejuni* on various processed meat products was conducted using FDA-iRISK 1.0. Inoculated processed meat products with 6.0 ± 0.5 log CFU/g of *C. jejuni* were vacuum packed and stored at 4, 10, 17, 24, 30, and 36 °C. Survival curves were fitted to the Weibull model to obtain the delta values of *C. jejuni* on various processed meat products. The most rapid death of *C. jejuni* was observed on dry-cured ham, followed by sausage without sodium nitrite. The results of semi-quantitative risk assessment indicate that dry-cured ham represented the lowest risk among all samples. *C. jejuni* on processed meats presented a greater risk at 4 °C than at 10 °C. The risk of ham was greater than the risk of sausage, regardless of type. Among all samples, the highest risk of *C. jejuni* was observed in round ham without sodium nitrite. Overall, our data indicates that risk of processed meat products due to *C. jejuni* is relatively low.

## 1. Introduction

*Campylobacter* continues to be the most commonly-reported gastrointestinal bacterial pathogen in humans worldwide [1]. Among the many species of *Campylobacter*, the one most commonly associated with human infection is *C. jejuni*, followed by *C. coli* [1,2,3]. Campylobacteriosis presents as 1–3 days of prodromal symptoms with fever, vomiting, and headaches followed by 3–7 days of watery or bloody diarrhea with abdominal pain. A great variation in severity of illness exists, ranging from mild disease to dehydration that might require hospitalization [4]. In addition, campylobacteriosis can induce neuropathies such as Guillain-Barré syndrome, Miller-Fisher syndrome, and Reiter syndrome [5,6,7,8,9,10].

In the EU, there were a total of 414 reported cases of *Campylobacter* infections in 2013, accounting for 8.0% of the total cases of foodborne illnesses [11]. A recent study in Canada reported an incidence of around 145,000 cases within a population of 32.5 million, placing *C. jejuni* as the third most frequent foodborne pathogen after norovirus and *Clostridium perfringens* [12]. In the US, there were also 6486 cases, 1080 hospitalizations, and 11 deaths caused by *Campylobacter* in 2014, according to the Foodborne Diseases Active Surveillance Network [13]. In Korea, there were 22 recorded cases of *Campylobacter* outbreaks and 805 hospitalizations in 2015 [14]. Although the incidence of *Campylobacter* outbreaks in Korea is low, caution must be taken since infection caused by *Campylobacter* has increased gradually. Thus, the risk of various meat products at retail markets due to *C. jejuni* contamination need to be evaluated.

According to the Korea Meat Industries Association [15] the volume of sales of processed meat increased from 10,963 tons in 1990 to 202,446 tons in 2013. The largest selling processed meats are ham products, of which a total of 63,336 tons were sold in 2013. Most of these processed meat products can be fit in one of the following four categories: cooked ham, dry-cured ham, luncheon meat, and fermented sausage [16]. The consumption of processed meat products such as ham has increased as a result of their convenience. Moreover, various types of ham, including garlic seasoned ham and nitrite-free ham, are available for sale in accordance with a trend of such food being seen as healthy and sophisticated [17]. Recently, dry-cured ham has also been developed and has gained great attention of consumers in Korea. In retail markets, vacuum packaging and storage under chilled conditions are widely used methods for storage of meat products [18]. The same packaging method and storage temperature is also applied to ham. It is well known that *C. jejuni* has susceptibility to various intrinsic and extrinsic factors, such as temperature, pH, water activity (Aw), and salinity [19]. It is also reported that low temperatures can enhance the survival ability of *C. jejuni* to these stressful conditions [20]. Moreover, many studies have indicated that refrigeration temperatures and vacuum packaging highly increase the risk of *C. jejuni* survival [21,22,23,24,25,26,27]. Accordingly, vacuum packaged and refrigerated ham products may be a good medium for the survival of *C. jejuni*. Thus, there is a need to identify and analyze the survival ability of *C. jejuni* on various processed meat products with different ingredients, which are very important data for the risk assessment of *C. jejuni* on various processed meat products.

FDA-iRISK^®^ is an interactive, web-based, comparative risk assessment tool made available by the U.S. Food and Drug Administration (FDA). It enables users to compare the risk of foodborne illness from microbial and chemical hazards and the public health impact of intervention and control measures [28]. iRISK was designed to calculate, through Monte Carlo simulation, the number of illness cases expected based on the contamination of the food by the hazard in question, the typical consumption pattern, and the dose-response relationship and then translates the number of cases into a public health metric to permit comparison of the public health burden across multiple food-hazard pairs [29].

In this work, we investigated the effect of garlic seasoning and nitrite on the survival of *C. jejuni* on vacuum-packaged ham at various temperatures. In addition, a semi-quantitative risk assessment of *C. jejuni* on various types of processed meat products was conducted using FDA-iRISK to compare the risk of *C. jejuni* on various types of processed meat products manufactured with or without garlic or nitrite at retail markets.

## 2. Materials and Methods

### 2.1. Bacterial Strains

Strains of *Campylobacter jejuni* (ATCC 33560 and NCTC 11168) were purchased from the Korean Culture Center of Microorganisms (KCCM, Seoul, Korea) and maintained at −80 °C in a form of beads stock (Viabank™, mwe, Corsham, Wiltshire, UK). For each experiment, stock for each bead of *C. jejuni* (ATCC 33560 and NCTC 11168) was inoculated into a 25 mL An Erlenmeyer flask containing 10 mL of sterile brucella broth (BD, Sparks, MD, USA) with 0.16% agar. A cocktail of *C. jejuni* cultures was placed in a microaerophilic chamber (DG250; Don Whitley Scientific, West Yorkshire, UK) with an atmosphere containing 5% oxygen, 10% carbon dioxide, and 85% nitrogen at 42 °C for 24 h. Viable cell counts of *C. jejuni* at the end of the incubation period ranged from 8.5 to 9.0 log CFU/mL. One mL from the stationary phase of an overnight culture was transferred into 9 mL of 0.1% sterilized peptone water (BD), and was serially diluted before inoculation into the sample. 

### 2.2. Preparation of Sample and Inoculation

In order to investigate a survival kinetics of *C. jejuni* on processed meat products, the following products were purchased from a local retail market: dry-cured ham, round ham with sodium nitrite, garlic seasoned ham with sodium nitrite, round ham without sodium nitrite, garlic seasoned ham without sodium nitrite, and sausage without sodium nitrite. They were all aseptically sliced into 10 g slices. Sliced samples were aseptically transferred to sterile Petri dishes and inoculated with 100 μL of the diluted cocktail mixture of *C. jejuni* strains using a sterile pipette for a target population of approximately 6.0 ± 0.5 log CFU/g. The inoculated samples were put into a polyethylene bag and vacuum packed (Fresh pack FM-03, Eiffel, Seoul, Korea). All inoculated and packaged samples were incubated at 4, 10, 17, 24, 30, and 36 °C (Incubator VS-120; Vision Scientific, Daejeon, Korea).

### 2.3. Enumeration of C. jejuni

At a selected sampling time based on the storage temperature of samples, each sample was homogenized (Stomacher, Interscience, Paris, France) with 90 mL of sterile 0.1% peptone water for 2 min. One milliliter of homogenized sample was diluted with 9 mL of 0.1% sterilized peptone water. 100 μL was spiral plated (Whitely automatic spiral plater, Don Whitley Scientific, West Yorkshire, UK) on selective media, mCCDA (modified charcoal cefoperazone deoxycholate agar) plates (Oxoid, Hampshire, UK) for *C. jejuni* in duplicate. All plates were incubated in a 42 °C chamber (DG250; Don Whitley Scientific) under microaerophilic conditions (5% O_2_, 10% CO_2_, and 85% N_2_) for 2–3 days. The colonies on duplicated plates of each sample were counted with an automated colony counter (Scan 1200, Interscience, Saint Nom, France). The mean of the duplicate plates was graphed at each sampling time to generate primary survival model for *C. jejuni* as a function of time. The same experiments were repeated twice. 

### 2.4. Primary Survival Modeling

Survival curves representing the viable counts (log CFU/g) of *C. jejuni* were graphed as a function of time and then iteratively fitted to the Weibull equation using a Gina FiT V 1.5 Program (Geeraerd and Van Impe Inactivation Model Fitting Tool) [30]. The model used was as follows:

Log10(N) = log10(N_0_) − ((t/delta)*p)

delta: treatment time for the 1st decimal reduction; p: shape; N_0_: log initial number of cells; t: time.

The Weibull model has two parameters; delta and p. The delta parameter represents the time of the first decimal reduction concentration for a part of the population. The Weibull distribution corresponds to a concave upward survival curve if p < 1 and concave downward if p > 1. If p = 1, the decrease is log-linear, which corresponds to a first-order decay reaction [31]. The goodness of fit of the data was evaluated based on the coefficient of determination (R^2^), which was also provided by Gina FiT V 1.5 Program. 

### 2.5. Secondary Survival Modeling

Delta values of the Weibull model were further applied to the Davey model as a function of temperature by using GraphPad PRISM V4.0 (GraphPad Software, San Diego, CA, USA) to assess the effect of temperature on delta values (secondary survival model). The Davey model used the following equation [32]:

Y = a + (b/T) + (c/T^2^)

Y: delta (hour); a, b, c: constant; T: temperature.

### 2.6. Water Activity (Aw), Salinity, and pH Analysis

To determine the water activity (Aw), 5 g samples were collected and measured at 25 °C using an Aqualab Lite (Decagon Devices, Inc. Pullman, WA, USA). pH was determined by homogenizing 10 g of each sample with 100 mL of distilled water and measuring each sample with a pH meter (IQ Scientific Instruments, Carlsbad, CA, USA). In addition, all samples were diluted 1:3 and salinity was measured with a salinity meter (Daekwang, Inc., Seoul, Korea).

### 2.7. Risk Comparison for Various Processed Meat Products Using FDA-iRISK

FDA-iRISK 1.0 was used to compare the risk of dry-cured ham, round ham with sodium nitrite, garlic seasoned ham with sodium nitrite, round ham without sodium nitrite, garlic seasoned ham without sodium nitrite, and sausage without sodium nitrite, which were stored at 4 °C and 10 °C. In this study, an FDA-iRISK template (Table 1) was configured to input various parameters, such as process model, consumption model, dose-response model, and burden of disease measure associated with health effects (e.g., losses in disability-adjusted life years (DALYs)) [29]. Since there was no data of the initial prevalence of *C. jejuni* on various kinds of processed meat products, the initial prevalence of *Campylobacter* spp. on ready-to-eat pork meat at the retail level [33] was used in this study. The minimum and maximum levels of the initial concentration were obtained from the experimental data of the present study to apply a worst-case scenario. In addition, the weight of each of the processed meat products in the market was entered as the initial unit mass. Depending on the sample, the initial unit mass value varied from 100 to 250 g. The process stage was at “decreased” which describes the removal or inactivation of the microbial hazard. This reflects the results from the primary survival modeling of experiments conducted in this study. To complete the consumption model, grams per eating occasion and eating occasions per year for ham products were specified as 30 g and 33.6 times, based on the report of Ministry of Health and Welfare [34], respectively. Since the consumption data for the dry-cured ham was not currently available, the proportion of a ham product that is consumed per eating occasion was used to estimate the grams per eating occasion for dry-cured ham. On average, consumers will eat one serving size (30 g) per 250 g unit mass. Using this proportion, 12 g (per 100 g unit mass) was estimated as grams per eating occasion for dry-cured ham. In addition, eating occasions per year for dry-cured ham was set as 3.3 times, which is a tenth of ham product. The low intake is due to its high salinity and low popularity within the market. A dose-response model was set using the Beta-Poisson model and a value of 0.024 was entered for α and 0.011 for β [35]. Finally, the input value to examine the effect on health was 0.03 DALYs per case [36]. 

### 2.8. Statistical Analysis

Experiments were replicated twice. Data was analyzed with the Statistical Analysis System SAS V 9.3 (SAS Institute Inc., Cary, NC, USA). The significance of the differences among the sample was determined by one-way ANOVA followed by Tukey’s range test for multiple comparisons at *p* < 0.05.

## 3. Results and Discussion

### 3.1. Measurement of Water Activity (Aw), Salinity, and pH on Processed Meat Products

The water activity (Aw), salinity, and pH values of various processed meat products tested in this study are shown in Table 2. The water activity of dry-cured ham was 0.852, which was the lowest value among the six samples. Unlike regular processed meat products, dry-cured ham passes through dry processing instead of heat treatment. Therefore, dry-cured ham has a low water activity, usually lower than 0.92 [37]. The optimal water activity level for the growth of *C. jejuni* has been shown to be 0.997 [19], and the water activity value of all tested samples was lower than the minimum water activity (0.987) for *C. jejuni* growth [19] except for sausage without sodium nitrite (0.992). Thus, all tested samples in this study, except sausage, had conditions unsuitable for *C. jejuni* growth. *C. jejuni* is also sensitive to salt concentrations above 1.5% [38]. The salinity values of dry-cured ham and round ham without sodium nitrite were 6.70 and 1.70, respectively, which falls within the sensitive salinity range of *C. jejuni*. The salt concentrations of round ham with sodium nitrite (1.35), garlic seasoned ham with sodium nitrite (1.10), garlic seasoned ham without sodium nitrite (1.40), and sausage without sodium nitrite (1.28) were all less than 1.5%, which may not be the significant level to prevent survival of *C. jejuni*.

According to Murphy *et al.* [39] the optimum pH range for *C. jejuni* growth is 6.5 to 7.5. The pH value of dry-cured ham (5.96 ± 0.010), round ham without sodium nitrite (6.38 ± 0.014), garlic seasoned ham without sodium nitrite (6.48 ± 0.013), and sausage without sodium nitrite (6.36 ± 0.013) was observed to be lower than the optimum range. However, the pH value of round ham with sodium nitrite (6.68 ± 0.013) and garlic seasoned ham with sodium nitrite (6.53 ± 0.021) was observed to be within the optimum range, indicating that addition of sodium nitrite increased the pH value of the product.

### 3.2. Primary Survival Model of C. jejuni on Processed Meat Products

**Figure 1:**
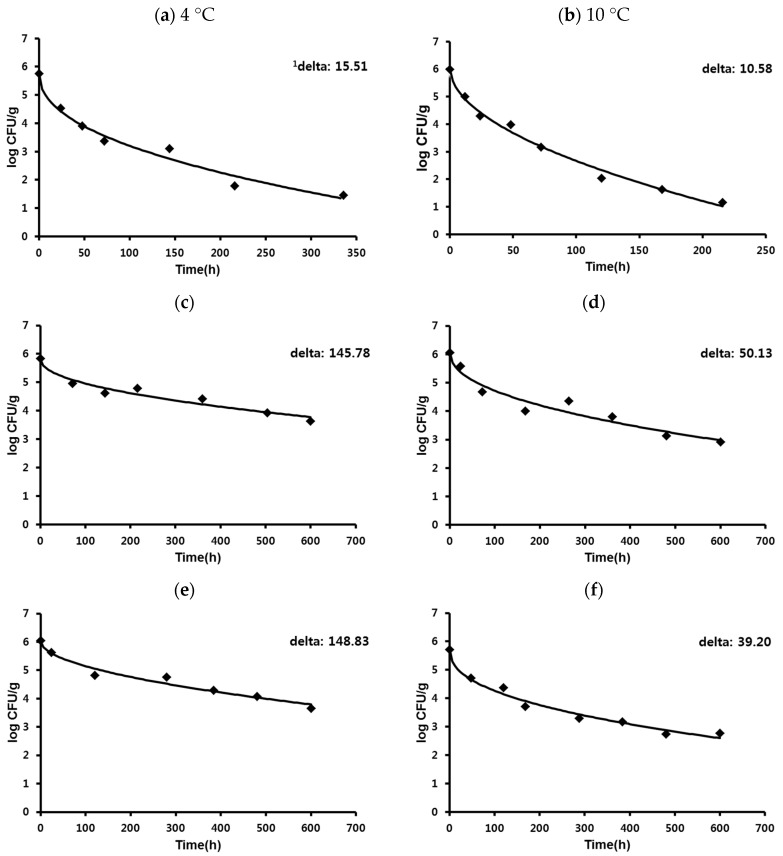
Primary survival models of *C. jejuni* on various processed meat products. (**a**) dry-cured ham at 4 °C; (**b**) dry-cured ham at 10 °C; (**c**) round ham with sodium nitrite at 4 °C; (**d**) round ham with sodium nitrite at 10 °C; (**e**) sausage without sodium nitrite at 4 °C; and (**f**) sausage without sodium nitrite at 10 °C. ^1^ Delta: treatment time for the first decimal reduction.

The kinetic data for the survival of *C. jejuni* on processed meat products under vacuum packaging condition at 4, 10, 17, 24, 30, and 36 °C were well fitted to the Weibull model with a high degree of goodness-of-fit (R^2^ > 0.90). The representative primary survival models for *C. jejuni* on processed meat products at 4 and 10 °C are shown in Figure 1. The rapid death of *C. jejuni* on dry-cured ham was observed and the populations of *C. jejuni* declined below the limit of detection within the expiration date (40 days) at both 4 °C and 10 °C. The time taken from the initial inoculation concentration to decrease to below the limit of detection was 15 and nine days at 4 °C (Figure 1a) and 10 °C (Figure 1b), respectively. At 36 °C, *C. jejuni* in dry-cured ham declined to 1 log CFU/g within 24 h (data not shown). These drastic reductions of *C. jejuni* may be affected by intrinsic characteristics of dry-cured ham, such as low water activity (0.852), low pH value (5.96), and high salinity (6.70%). On the other hand, the survival trend of *C. jejuni* on sausage was similar to the processed ham in the present study. Especially, the survival kinetics of *C. jejuni* in round ham with sodium nitrite (Figure 1c,d) was most similar to those in sausage without sodium nitrite (Figure 1e,f), indicating that addition of sodium nitrite did not affect the survival kinetics of *C. jejuni* in processed ham and sausage. No significant differences in the values of Aw, pH, and salinity were observed between these two samples in the current study. During 600 h (25 days) of storage, *C. jejuni* in sausage without sodium nitrite significantly survived better at 4 °C than 10 °C, indicating the effect of temperature on survival ability of *C. jejuni* (Table 3).

In this study, *C. jejuni* survived well at refrigeration temperatures, especially at 4 °C, than at higher temperatures, regardless of the sample type tested. Similar to the results of the current study, other studies have also shown that *C. jejuni* can survive better at 4 °C than at ambient temperatures in different environments, including different food matrices [21,22,23,24,25,26,27]. Stintzi and Whitworth [40] described an adaptive response in *C. jejuni* to low temperature by modifications in gene expression to explain how the bacteria can survive in this condition for long periods of time. Moreover, Hazeleger *et al.* [41] reported that protein synthesis of *C. jejuni* takes place at low temperatures, even at 4 °C, which is far below the minimal growth temperature, is able to respire and survive for a long period.

However, the survival ability of *C. jejuni* on processed meat products at 36 °C in our study was different from other previous studies. Blankenship and Craven [42] observed that *C. jejuni* in ground chicken meat grows during the first four days at 37 °C. After four days, *C. jejuni* showed a gradual 1 log decline by 17 days of storage. In the study of Burnette and Yoon [22], there was no growth or death of *C. jejuni* on cooked, aerobic packaged chicken breast patties at 37 °C for 25 h. Additionally, Park, Ro, Jo, Park, and Yoon [27] found that *C. jejuni* on vacuum packaged chicken breast at 36 °C decreased only 1.7 log CFU/g at 150 h of storage. In contrast to these previous studies, the current study showed that *C. jejuni* in vacuum packaged ham products declined about 4 log CFU/g within 24 h at 36 °C, regardless of sample type. This difference is thought to be a result of differences in the intrinsic factors between processed ham products and unprocessed chicken breast meat as food matrices. Consequently, the population of *C. jejuni* on processed meat products under vacuum packaging declined faster as temperature increased but survived well at refrigeration temperature. Thus, special attention should be paid to the manufacturing process of ham products to prevent contamination with *C. jejuni* since the infectious dose of *C. jejuni* is low [3].

### 3.3. Effect of Temperature and Seasoning on the Survival Ability of C. jejuni in Processed Meat Products

**Figure 2:**
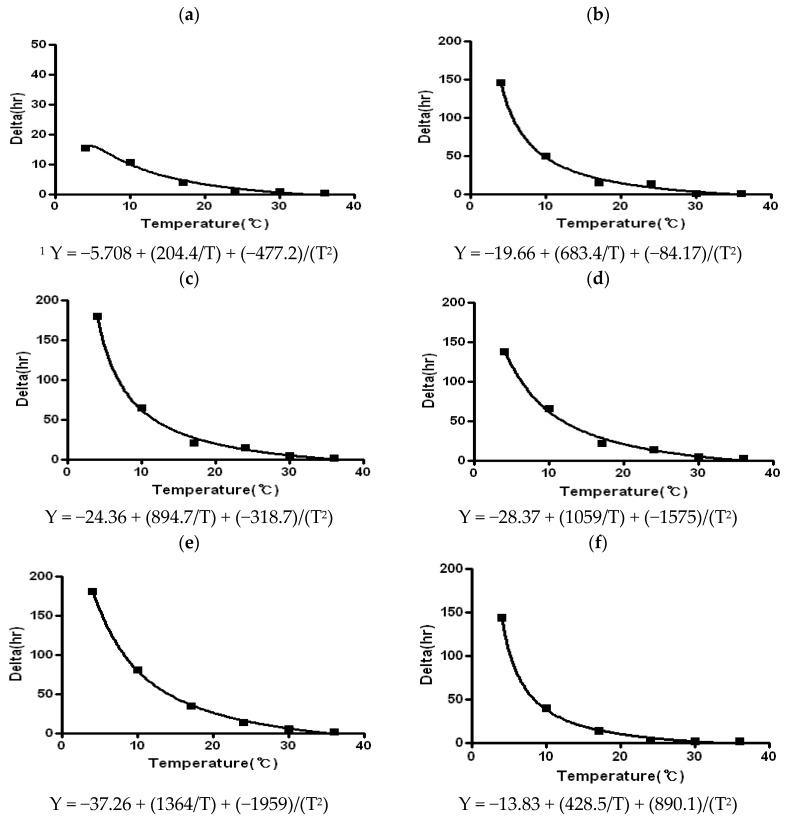
Secondary survival model of *C. jejuni* on vacuum packaged processed meat products as a function of temperature. (**a**) Dry-cured ham; (**b**) round ham with sodium nitrite; (**c**) garlic seasoning ham with sodium nitrite; (**d**) round ham without sodium nitrite; (**e**) garlic seasoning ham without sodium nitrite; and (**f**) sausage without sodium nitrite. ^1^ Davey model: Y = a + (b/T) + (c/T^2^), T: temperature.

Table 3 shows the survival kinetics (delta) of *C. jejuni* on various processed meat products as a function of temperature. Secondary survival models were also developed to describe the effect of temperature on the primary model parameter (delta) (Figure 2). The delta parameter represents the time of the first decimal reduction concentration for a part of the population.

When the effect of temperature on the survival kinetics of *C. jejuni* on processed meat products was compared, the higher temperature tended to have a lower delta value in all sample types, indicating that higher temperatures increased the death rate of *C. jejuni* in processed meat products. The greatest survival of *C. jejuni* was observed in all samples at 4 °C. Significant (*p* < 0.05) differences of delta values were observed at refrigeration temperatures between 4 and 10 °C, regardless of sample types. The delta values at 4 °C were two or three times higher than those at 10 °C, except for the dry-cured ham. The delta values of dry-cured ham were 15.51 h at 4 °C and 10.58 h at 10 °C, which are significantly (*p* < 0.05) lower compared to the other samples. This result indicates that dry-cured ham with a low water activity, low pH and high salinity has poor conditions for *C. jejuni* survival. As such, *C. jejuni* could not survive and declined within a very short time in the dry-cured ham even at refrigeration temperatures (Figure 1a,b). Although delta values of dry-cured ham were not significantly different at 17 °C, 24 °C, 30 °C, and 36 °C, the delta value of *C. jejuni* on dry-cured ham decreased more rapidly as temperature increased.

Except dry-cured ham, there were no significant differences in delta values among all samples at 4 °C, but significant differences in delta values were observed among the samples at 10 °C (Table 3). The lowest delta value was observed in dry-cured ham (10.58 h), followed by sausage without sodium nitrite (39.20 h), round ham with sodium nitrite (50.13 h), garlic seasoning ham with sodium nitrite (65.14 h), round ham without sodium nitrite (65.26 h), and garlic seasoning ham without sodium nitrite (81.03 h). In all cases, no significant differences in delta values were observed between 17 °C and 24 °C or between 30 and 36 °C in the same sample. Overall, the delta value of *C. jejuni* on ham with sodium nitrite was relatively low compared to that of ham without sodium nitrite, indicating that addition of sodium nitrite may affect the death rate of *C. jejuni* on ham. However, there was no significant difference of delta value between round ham with sodium nitrite (145.78 h) and round ham without sodium nitrite (137.70 h), as well as between garlic seasoned ham with sodium nitrite (179.20 h) and garlic seasoned ham without sodium nitrite (181.12 h) at 4 °C. This result may suggest that addition of sodium nitrite in ham does not have a strong antimicrobial effect against *C. jejuni* at 4 °C, while addition of sodium nitrite prevents survival of *C. jejuni* in ham at 10 °C. However, the delta values of *C. jejuni* were not significantly affected by sodium nitrite as temperature increased.

Sodium nitrite is commonly used in meat processing for providing the cured color, flavor, and microbiological safety [43]. The concentration of sodium nitrite is limited to 70 ppm in Korea whereas the U.S. limits the concentration of sodium nitrite to 156 ppm. Overall, 70 ppm of sodium nitrite added to the ham may not be sufficient to prevent the survival of *C. jejuni*, especially at 4 °C and at ambient temperature. Additionally, the type of *C. jejuni* strain used in our study may have affected the results. Uradziński and Szteyn [44] studied the effect of sodium nitrite, sodium chloride, and ascorbic acid on three *C. jejuni* strains inoculated in ground pork meat. One of the strains was resistant to all of the chemical preservatives tested. On the other hand, the other two strains were sensitive to sodium nitrite and ascorbic acid. As such, these studied indicated that the sensitivity to sodium nitrite differs depending on the *C. jejuni* strain. Based on this result, strains of *C. jejuni* (ATCC 33560 and NCTC 11168) used in this study were considered to be relatively less sensitive to sodium nitrite. Not only may *C. jejuni* strain type be having an effect but also the respiratory ability of *C. jejuni* could have affected the results at 4 °C. In fact, several studies have shown that that nitrite supports the respiration of *C. jejuni* [45,46]. In addition, the survival of *C. jejuni* on processed meat products is controlled by the interaction of temperature, pH, Aw, salinity, oxygen content, and other food additives, amongst other factors.

Among the samples, the slowest reduction of *C. jejuni* was also observed in garlic seasoned ham without sodium nitrite at all temperatures. There are many reports on the antibacterial activity of garlic extract and allicin [47,48,49,50,51,52,53,54,55,56,57]. Those studies proved that garlic is effective against many pathogens such as *Escherichia coli*, *Bacillus cereus*, *Staphylococcus aureus*, *Vibrio parahaemolyticus, Listeria monocytogenes, Salmonella typhi,* and others. In particular, studies conducted by Lu, Rasco, Jabal, Aston, Lin, and Konkel [57] and Ross, O’Gara, Hill, Sleightholme and Maslin [49] confirmed that garlic extract inhibits the survival of *C. jejuni*. However, results of the present study suggest that there is no strong antimicrobial effect on *C. jejuni* by the garlic extract added to the ham in this study. The amount of garlic extract added in commercial ham may not be sufficient to prevent the survival of *C. jejuni*.

### 3.4. Risk Comparison of Various Processed Meat Products Using FDA-iRisk

Based on the data inputs for *C. jejuni* with various processed ham products, FDA-iRISK generated risk estimates through Monte Carlo simulations for each of the six different products stored at 4 °C and 10 °C (Table 4). The computations were conducted using the Analytica Decision Engine (Lumina Decision Systems, Los Gatos, CA, USA) [29]. All entered scenarios in the current study were converged with the Monte Carlo simulation. The results include the total number of illnesses, mean risk of illness, per eating occasion of the consumer, and total DALYs per year. The risk of each product was compared relatively according to the storage temperature, added ingredients, and the type of sample in the current study. At 4 °C, total number of illnesses of dry-cured ham was 0.000692, the lowest value among the samples. The mean risk of illness indicates the average probability of illness from one serving or eating occasion. Similar to the results for total number of illnesses, dry-cured ham had the lowest value of mean risk of illness, 0.000210 (2.1 cases per ten thousand servings). Combining the mean risk of illness output with the number of servings per year, the expected annual number of cases was calculated and subsequently translated into annual DALYs loss [29]. The value of total DALYs per year in dry-cured ham was 0.0000208, which was the lowest value followed by sausage without sodium nitrite (0.000320), round ham with sodium nitrite (0.000329), garlic seasoned ham with sodium nitrite (0.000335), garlic seasoned ham without sodium nitrite (0.000346), and round ham without sodium nitrite (0.000348). In other words, dry-cured ham represented the lowest risk among six kinds of samples tested in the present study. The risk of sausage without sodium nitrite was lower than the risk of the remaining four types of ham. Among the ham samples, the risk of ham with sodium nitrite was lower than the risk of ham without sodium nitrite. Recently “no nitrite added” processed meat products have increased significantly in the retail market due to the consumer preference for “additive, preservative-free products”. Thus, the safety and shelf life of various kinds of processed meat products without sodium nitrite must be carefully evaluated at retail market.

The results at 10 °C showed the same tendency as with the previous result at 4 °C. Total DALYs per year of dry-cured ham was 0.0000201, the lowest among the six samples, and a lower risk than the annual DALYs at 4 °C. However, risk of *C. jejuni* in processed meat products, even at 10 °C, should not be overlooked since the infectious dose of *C. jejuni* is low. This result also indicates that ham without sodium nitrite has a higher risk than ham with sodium nitrate at 10 °C. In other words, sodium nitrite inhibits the survival of *C. jejuni* slightly. However, the risk reduction with the garlic seasoning was not clearly observed at both 4 and 10 °C. Moreover, our experiments suggest that sausage has a lower risk than ham (except dry-cured ham), regardless of the presence of sodium nitrite. This result may come from the differences of the food matrix between sausage and ham.

## 4. Conclusions

Overall, the survival kinetics of *C. jejuni* was slightly different on the various processed ham products due to the differences in their intrinsic factors and food matrices, such as meat type, additives, and other factors. *C. jejuni* survived better within the shelf life of processed meat products at 4 °C than at 10 °C. 70 ppm of sodium nitrite and garlic seasoning added to the ham was not sufficient to prevent the survival of *C. jejuni* in the present study. Although risk of processed meat products due to *C. jejuni* is relatively low at retail market, cross-contamination with *C. jejuni* should be prevented during and after the manufacturing of processed meat products.

With iRISK, the relative risk of each product was compared according to the storage temperature, added ingredients, and the type of sample in the current study. At 4 °C, dry-cured ham had the lowest value of mean risk of illness, 0.000210 (2.1 cases per ten thousand servings) among the samples, followed by sausage without sodium nitrite (3.18 cases per ten thousand servings). The value of total DALYs per year was higher at 4 °C than at 10 °C, also indicating that there is a greater risk of *C. jejuni* on various processed meat products at 4 °C than at 10 °C. Although use of the iRISK model is relatively simple and easy due to the built-in model framework and math calculations, the user must enter data based on the literature or experimental study and must have the knowledge to create scenarios for process models, consumption models, dose-response relationships, and health outcomes, *etc.*

## Figures and Tables

**Table 1:** Model inputs for food-hazard scenarios in FDA-iRISK 1.0.

Element of Risk Scenario	*C. jejuni* in Processed Meat Products, Total Population		
	Input Parameter, iRISK Template	Model Input	References
Food	Processed meat products	Description	
Hazard	*C. jejuni*	Description	
Process model	Initial prevalence	0.001	Mataragas *et al*. (2008) [31]
	Initial concentration	A: Uniform (5.75, 5.88/6.00, 6.00) log CFU B: Uniform (5.85, 5.98/6.06, 6.06) log CFU C: Uniform (5.86, 5.88/6.14, 6.15) log CFU D: Uniform (5.99, 6.02/6.05, 6.13) log CFU E: Uniform (5.92, 6.02/6.15, 6.24) log CFU F: Uniform (5.62, 6.04/5.29, 5.72) log CFU	
	Initial unit mass	A: 100 g, B: 250 g, C: 210 g, D: 250 g, E: 160 g, F: 350 g	
	Process stage 1: storage at 4 and 10 °C, decrease	(a): Uniform (4.30, 4.88/4.85, 5.00) log CFU (b): Uniform (2.17, 2.22/3.15, 3.31) log CFU (c): Uniform (1.95, 2.04/2.94, 3.16) log CFU (d): Uniform (1.68, 1.88/2.59, 2.62) log CFU (e): Uniform (1.67, 1.94/2.83, 2.97) log CFU (f): Uniform (2.32, 2.38/2.68, 2.94) log CFU	
Consumption model	Grams per eating occasion	A: 12 g/B,C,D,E,F: 30 g	
	Eating occasions per year	A: 3.3/B,C,D,E,F: 33.6	Ministry of Health and Welfare and Centers for Disease Control and Prevention (2011) [34]
Dose-response model	Beta-Poisson model	α = 0.024; β = 0.011	Teunis *et al*. (2005) [35]
Health effects	DALY template (Campylobacteriosis)	0.03 DALYs per case	Kemmeren *et al*. (2006) [36]

Initial concentration input: minimum, maximum of 4 °C/minimum, maximum of 10 °C. Process stage input: minimum, maximum of 4 °C/minimum, maximum of 10 °C. (a) dry-cured ham; (b) round ham with sodium nitrite; (c) garlic seasoning ham with sodium nitrite; (d) round ham without sodium nitrite; (e) garlic seasoning ham without sodium nitrite; and (f) sausage without sodium nitrite.

**Table 2:** The water activity (Aw), salinity and pH values of processed meat products.

Sample	Dry-Cured Ham	Round Ham with Sodium Nitrite	Garlic Seasoning Ham with Sodium Nitrite	Round ham without Sodium Nitrite	Garlic Seasoning Ham without Sodium Nitrite	Sausage without Sodium Nitrite
Aw	0.852 ± 0.0022	0.972 ± 0.0029	0.980 ± 0.0017	0.983 ± 0.0026	0.984 ± 0.0024	0.992 ± 0.0017
Salinity (%)	6.70 ± 0.38	1.35 ± 0.30	1.10 ± 0.20	1.70 ± 0.12	1.40 ± 0.23	1.28 ± 0.15
pH	5.96 ± 0.010	6.68 ± 0.013	6.53 ± 0.021	6.38 ± 0.014	6.48 ± 0.013	6.36 ± 0.013

Means (*n* = 4) ± SD.

**Table 3:** Kinetic parameters (delta) for *C. jejuni* survival on various processed meat products as a function of temperature.

Sample	Delta ^1^ (Hour)					
	4 °C	10 °C	17 °C	24 °C	30 °C	36 °C
Dry-cured ham	^B^ 15.51 ^a^	^E^ 10.58 ^a,b^	^C^ 3.84 ^b,c^	^B^ 1.20 ^c^	^B^ 0.89 ^c^	^D^ 0.31 ^c^
Round ham with sodium nitrite	^A^ 145.78 ^a^	^C^ 50.13 ^b^	^B,C^ 14.27 ^c^	^A^ 13.21 ^c^	^B^ 0.91 ^c^	^C,D^ 0.65 ^c^
Garlic seasoning ham with sodium nitrite	^A^ 179.20 ^a^	^B^ 65.14 ^b^	^A,B,C^ 20.26 ^c^	^A^ 14.54 ^c,d^	^A^ 5.13 ^d,e^	^A,B^ 1.99 ^e^
Round ham without sodium nitrite	^A^ 137.70 ^a^	^B^ 65.25 ^b^	^A,B^ 21.82 ^c^	^A^ 13.79 ^c^	^A^ 4.84 ^c^	^A^ 2.81 ^c^
Garlic seasoning ham without sodium nitrite	^A^ 181.12 ^a^	^A^ 81.03 ^b^	^A^ 34.49 ^c^	^A^ 14.08 ^d^	^A^ 5.81 ^d^	^A,B,C^ 1.71 ^d^
Sausage without sodium nitrite	^A^ 148.83 ^a^	^D^ 39.20 ^b^	^B,C^ 13.53 ^b,c^	^B^ 2.65 ^c^	^B^ 1.52 ^c^	^B,C,D^ 1.35 ^c^

^1^ Delta: treatment time for the first decimal reduction; ^a–e^ Dissimilar superscripts in the same row denote significant difference (*p* < 0.05); ^A–E^ Dissimilar superscripts in the same column denote significant difference (*p* < 0.05).

**Table 4:** FDA-iRISK output: risk ranking of population health burden across multiple processed meat products stored at 4 and 10 °C.

Sample	Total No. of Illnesses	Mean Risk of Illness	Per Eating Occasions or Consumer	Total DALYs per Year
Dry-cured ham	0.000692 (0.000671) ^1^	2.10 × 10^−4^ (2.03 × 10^−4^)	6.29 × 10^−6^ (6.10 × 10^−6^)	0.0000208 (0.0000201)
Sausage without sodium nitrite	0.0107 (0.00965)	3.18 × 10^−4^ (2.87 × 10^−4^)	9.53 × 10^−6^ (8.62 × 10^−6^)	0.000320 (0.000290)
Round ham with sodium nitrite	0.0110 (0.00983)	3.27 × 10^−4^ (2.93 × 10^−4^)	9.80 × 10^−6^ (8.78 × 10^−6^)	0.000329 (0.000295)
Garlic seasoning ham with sodium nitrite	0.0112 (0.0102)	3.32 × 10^−4^ (3.03 × 10^−4^)	9.97 × 10^−6^ (9.09 × 10^−6^)	0.000335 (0.000305)
Round ham without sodium nitrite	0.0116 (0.0107)	3.45 × 10^−4^ (3.18 × 10^−4^)	1.04 × 10^−5^ (9.53 × 10^−6^)	0.000348 (0.000320)
Garlic seasoning ham without sodium nitrite	0.0115 (0.0104)	3.43 × 10^−4^ (3.11 × 10^−4^)	1.03 × 10^−5^ (9.32 × 10^−6^)	0.000346 (0.000313)

^1^ (⋯): data at 10 °C.
